# Management of Rare Temporomandibular Joint Cysts with Intracranial Extension: A Case Series and Literature Review

**DOI:** 10.1055/a-2620-3584

**Published:** 2025-06-17

**Authors:** Lindsey Jackson, Jacob Poynter, Maryam Rahman, Tara Massini, Si Chen

**Affiliations:** 1 Department of Otolaryngology—Head and Neck Surgery, University of Florida College of Medicine, Gainesville, Florida, United States; 2 Department of Neurosurgery, University of Florida College of Medicine, Gainesville, Florida, United States; 3 Department of Radiology, University of Florida College of Medicine, Gainesville, Florida, United States

**Keywords:** temporomandibular joint, TMJ cyst, intracranial extension, intracranial abscess, middle cranial fossa, MCF defect

## Abstract

**Introduction:**

Temporomandibular joint (TMJ) cysts extending through the skull base into the middle cranial fossa (MCF) are rare, with limited data on clinical progression and treatment. This study retrospectively analyzed three cases of TMJ cysts with MCF extension managed by a multidisciplinary team. Clinical presentation, imaging, surgical resection, outcomes, and a literature review are presented.

**Case Presentations:**

Three patients presenting with otalgia and TMJ tenderness were found to have intracranial cysts communicating with the TMJ. Two patients had been transferred with suspected intracranial abscesses; one presented for workup of headache and trigeminal neuralgia. All three demonstrated elevation of inflammatory markers. Two patients had TMJ aspiration, notable for leukocytosis and crystalline deposition, another had frank purulence. One patient demonstrated pneumocephalus within the cyst on imaging. The intracranial cysts ranged from 1.2 to 3.3 cm in maximum diameter, with their bony defects ranging from <1 to 4 mm. Two patients underwent craniotomy, cyst resection, and repair of the middle fossa defect, while the third opted for observation. Pathology of the white gelatinous fluid within the two resected growths demonstrated benign cysts.

**Conclusion:**

TMJ cysts with intracranial extension, while rare, require careful differentiation from intracranial abscesses. Surgical urgency may be indicated in cases demonstrating clinical signs of infection. Additionally, TMJ cysts with intracranial extension benefit from surgical removal and skull base repair to relieve symptoms and prevent future complications.

## Introduction

Benign cystic lesions of the temporomandibular joint (TMJ) that extend intracranially into the middle cranial fossa (MCF) are rare, with only three cases previously reported in the literature, but can present unique diagnostic and therapeutic challenges. 1 2 3 4 5 Diagnostically, these cysts can be difficult to differentiate from other intracranial lesions such as tumors or abscesses. 1 2 4 5 Given the extremely thin roof of the TMJ glenoid fossa, this area of the middle fossa floor is particularly vulnerable to erosion. 5 6 7 Therefore, management of these cysts prevents complications such as cerebrospinal fluid (CSF) leakage, meningitis, and cortical injury. 4 5 6 Timely diagnosis and intervention are essential to ensure these patients achieve the most favorable outcomes.

Patients with TMJ cysts extending to the MCF typically present with nonspecific symptoms such as otalgia, trismus, and preauricular tenderness. 3 4 5 6 These symptoms are also occasionally accompanied by cranial nerve deficits. 1 2 4 5 However, the number of documented cases with such intracranial spread remains limited, highlighting the need for further study on their clinical progression and management. 1 2 3 4 5 This study presents the management of three cases of TMJ cysts with intracranial extension, detailing clinical presentation, imaging features, and surgical approaches, as well as insights from a comprehensive literature review. By contributing to the limited data on this condition, we aim to inform future diagnostic and therapeutic approaches. Our findings emphasize the importance of multidisciplinary evaluation and tailored surgical management to optimize outcomes and mitigate the risk of severe complications.

## Case Presentations

### Case 1

A 79-year-old woman with a past medical history of hypertension, coronary artery disease, and diabetes mellitus presented to an outside hospital with a several-day history of dizziness, syncope, multiple falls, slurred speech, intermittent left-sided otalgia, bilateral TMJ pain, bilateral lower extremity weakness, and urinary incontinence. She denied any fevers, chills, nausea, vomiting, headaches, neck pain, neck stiffness, changes in vision, or changes in hearing. Initial physical exam demonstrated bilateral weakness of the lower face, bilateral weakness of the lower extremities, and tongue protrusion to the right but was otherwise unremarkable. The patient was started on broad-spectrum intravenous (IV) antibiotics at that time.

**Fig. 1:**
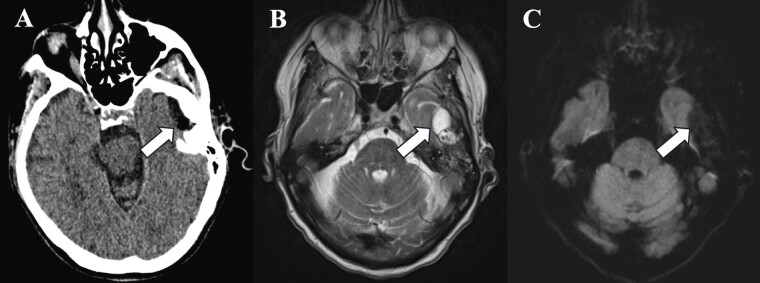
Case 1 axial postcontrast CT ( **A** ), T2-weighted MR ( **B** ), and diffusion-weighted MR ( **C** ) images. These images show a left MCF cyst with rim enhancement (arrow A), small foci of air within the intracranial cyst (arrow B), and no water restriction (arrow C) to suggest a bacterial abscess. CT, computed tomography; MR, magnetic resonance.

**Fig. 2:**
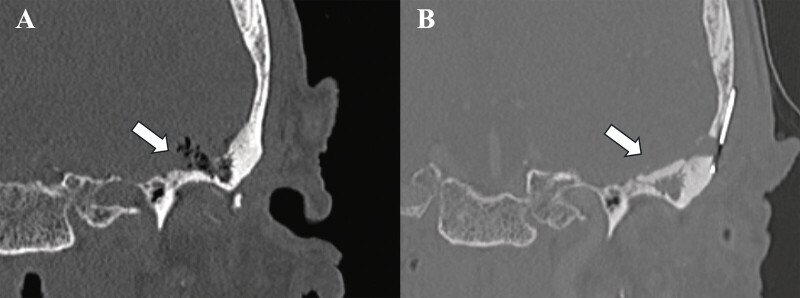
Case 1 coronal CT images before ( **A** ) and after ( **B** ) surgical repair. Note there was air within the intracranial cyst at presentation (arrow A). The skull base defect was repaired with bone cement (arrow B). CT, computed tomography.

Laboratory workup was notable for elevated high-sensitivity C-reactive protein (CRP), erythrocyte sedimentation rate (ESR), white blood cell (WBC), and blood glucose level. Computed tomography (CT) head without IV contrast revealed a collection of gas and fluid at the base of the left temporal lobe, which was contiguous with an osseous defect at the roof of the left TMJ ( Figs. 1A and 2A ). The patient was transferred urgently from the outside hospital for suspicion of an intracranial abscess. Neurosurgery, ENT, and Oral and Maxillofacial Surgery (OMFS) saw the patient that day and recommended magnetic resonance imaging (MRI) for further evaluation. MRI showed active synovitis in the left TMJ with communication to the left MCF, where there was a multiloculated collection of epidural fluid, as well as adjacent dural enhancement and a mild regional mass effect ( Fig. 1B ). No adjacent edema in the overlying brain or water restriction within the fluid was observed.

Upon review of the MRI, the patient was scheduled for surgical intervention at the earliest possible time. She underwent a left middle fossa craniotomy with neurosurgery and otolaryngology for cyst removal, and the skull base was repaired with a fascia lata graft from the left leg.

Pathology demonstrated fibrous tissue compatible with a synovial cyst with focal eosinophilic infiltrate. Postoperative CT showed no residual enhancing collection in the MCF, no evidence of temporal lobe encephalocele, erosive changes extending from the left TMJ to the posterosuperior direction, and persistent left TMJ effusion ( Fig. 2B ). About one month after her surgery, outpatient follow-up showed well-healed incisions, resolution of dizziness and syncope, and restoration of facial muscle symmetry. She continued to have left TMJ pain, for which she was referred to a specialized orthodontic clinic.

### Case 2

A 44-year-old woman presented with recurrent episodes of headache, nausea, insomnia, and severe left TMJ pain. She denied syncope, unilateral weakness, changes in vision, or any recent trauma. No abnormalities were noted on the physical exam. Initial complete blood count (CBC) and basic metabolic panel were within normal limits except for an elevated CRP and slightly elevated ESR.

**Fig. 3:**
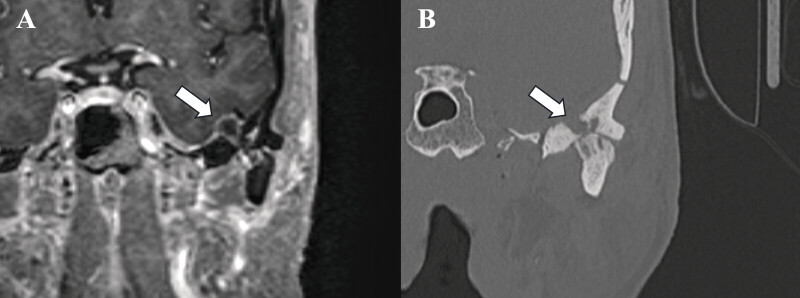
Case 2 coronal postcontrast T1-weighted MR image ( **A** ) and coronal CT ( **B** ). These images show an intracranial rim-enhancing cyst in the left MCF with tunneling through the bone and communication with the TMJ (arrow A). Note advanced joint space loss and other subchondral cyst formation in the mandibular condyle and condylar fossa (arrow B). CT, computed tomography; MR, magnetic resonance; TMJ, temporomandibular joint.

An MRI of the brain revealed a small extra-axial fluid collection over the undersurface of the left temporal lobe communicating inferiorly through the skull base with the TMJ, with overlying dural enhancement and bone erosion in the mandibular condyle, but without water restriction to confirm abscess ( Fig. 3A ). The patient was referred to Neurosurgery and OMFS, who recommended surgical intervention due to the potential destructive nature of the cyst.

Preoperative CT showed chronic erosive monoarticular disease of the left TMJ with a possible secondary synovial cyst extending extradurally into the floor of the left MCF ( Fig. 3B ). The patient underwent a left temporal craniotomy for cyst evacuation, repaired with left lower extremity fascia lata graft, and TMJ arthroplasty. She tolerated the procedure well with no complications. The surgical pathology report showed a benign cyst with areas of mucoid degeneration and focal calcium pyrophosphate dihydrate crystal deposition. At an outpatient follow-up visit about 1 month after her surgery, the patient had well-healed incisions and resolution of symptoms.

### Case 3

A 79-year-old man with a past medical history of bilateral sensorineural hearing loss, chronic obstructive pulmonary disease, and diabetes mellitus presented with acute onset pain in his right ear and jaw, which was severe enough to limit his food intake. He denied any headaches, nuchal rigidity, fevers, or photophobia. Laboratory workup was notable for high-sensitivity CRP and ESR but was otherwise unremarkable including WBC. The patient was transferred urgently from an outside hospital for suspicion of otitis externa with mastoiditis and a possible epidermal abscess.

**Fig. 4:**
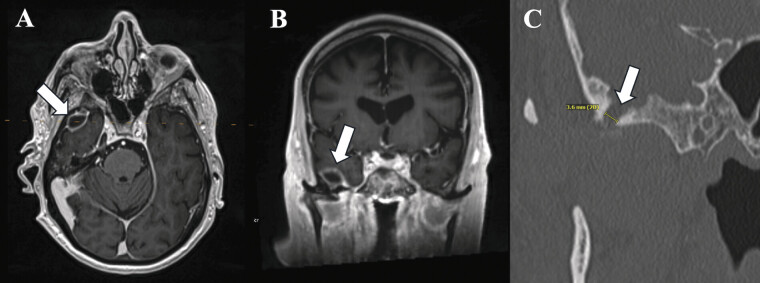
Case 3 postcontrast T1-weighted MR images in axial ( **A** ) and coronal ( **B** ) planes and coronal CT ( **C** ). These images show a rim-enhancing intracranial collection in the right MCF (arrows A and B) with a small bone defect in communication with the TMJ (arrow C). This patient did not undergo surgical repair. CT, computed tomography; MCF, middle cranial fossa; MR, magnetic resonance; TMJ, temporomandibular joint.

CT with IV contrast of the right temporal bone revealed a synovial cyst extending from the TMJ through the temporal bone into the epidural space in the anterior MCF, severe chronic bony remodeling of the TMJ, a 3.6-mm defect in the floor of the TMJ joint, and opacification involving the right middle ear and right mastoid air cells ( Fig. 4C ). MRI of the brain for further evaluation showed findings consistent with severe degenerative changes of the right TMJ with synovial cyst extension into the right MCF and without evidence of diffusion restriction to suggest abscess ( Fig. 4A, B ). It also showed findings consistent with minimal right-sided otomastoiditis. Synovial fluid was then aspirated from the right TMJ, which demonstrated frank purulence and grew methicillin-resistant *Staphylococcus aureus* .

Due to multiple comorbidities, this patient chose to continue with medical therapy and observation instead of middle fossa repair. Infectious disease was consulted and recommended a 10-day regimen of clindamycin 150 mg three times daily and levofloxacin 500 mg daily. About 2 weeks later at an outpatient follow-up visit, his TMJ pain and symptoms of otomastoiditis had resolved.

## Discussion

### Brief Literature Review

Our literature review reveals only 30 cases of TMJ cysts, pointing to a very low prevalence, underdiagnosis, underreporting, or most likely a combination of these factors. 1 2 3 4 5 Prior to this study, only three of these reported cases had demonstrated intracranial extension into the MCF. 1 2 6 8 9 10 Only one of those cases was initially treated with simple aspiration. That patient's cyst recurred less than 3 months later and was then treated with surgical resection. The two patients initially treated with surgical resection showed no evidence of recurrence, with follow-up lasting 2 years for 1 and 5 years for the other.

Possible complications of these otherwise benign cysts underscore the need to comprehensively explore the nuances of diagnosing and managing these cases. 4 5 6 The three cases presented in this study provide critical insights into the differentiation of TMJ cysts from other pathologies, the necessity for surgical intervention in cases with bony erosion, and the spectrum of presenting symptoms and outcomes. This discussion seeks to enhance awareness of this potential diagnosis among surgeons, aiming to inform and improve future surgical decision-making.

### Pathophysiology

TMJ cysts with intracranial extension are thought to develop through a complex inflammatory process. As with extracranial TMJ cysts, this process often begins with chronic damage to the TMJ synovial membrane, leading to an inflammatory cascade led by cytokines such as interleukin-1beta (IL-1β) and tumor necrosis factor-alpha. 11 The resulting degeneration of collagen in the TMJ capsule allows fluid-filled cysts to form, and in the case elevated intra-articular pressure, these cysts may herniate outside of the synovium.

### Pathological Differentiation

One of the most significant diagnostic challenges in evaluating TMJ cysts with intracranial extension is distinguishing them from other potentially erosive processes. Synovitis, degenerative cysts, septic arthritis, synovial chondromatosis, giant cell tumors, and other primary tumors of cartilage or bone in this area can all have overlapping and nonspecific clinical features. 1 2 4 5 However, their underlying etiologies and management strategies differ substantially, making accurate differentiation imperative.

Imaging often plays a pivotal role in this process. TMJ cysts, as demonstrated in all three cases in this study, typically appear as well-circumscribed, fluid-filled lesions with rim enhancement and central fluid on MRI. 1 2 4 5 Importantly, the absence of diffusion restriction on MRI diffusion-weighted imaging (DWI) sequences is a hallmark feature of cysts, contrasting with abscesses, which typically exhibit restricted diffusion due to their high cellularity and viscous pus content. 4 5 6

The importance of imaging in differentiation is well illustrated by Case 3. Despite initial concerns for an abscess based on clinical findings of severe TMJ pain and purulent aspiration, MRI findings were more consistent with a cystic process. The lack of restricted diffusion on DWI and the clear continuity of the lesion with the TMJ suggested a benign cyst rather than an abscess. This case also highlights that while imaging can strongly suggest a benign or infectious process, definitive diagnosis may sometimes rely on more invasive testing, such as needle aspiration or biopsy.

Accurate differentiation between TMJ cysts and abscesses has profound implications for patient management. Abscesses often require urgent drainage and prolonged antibiotic therapy to prevent complications such as meningitis, cerebral abscess, or sepsis. 4 5 6 Conversely, TMJ cysts, particularly those without evidence of infection or significant symptoms, may be amenable to conservative management or elective surgical intervention. 3 6

### Surgical Intervention

The thin bone of the TMJ glenoid fossa, averaging less than 1 mm, is particularly vulnerable to erosion. 5 6 7 This can create direct communication between the TMJ and the MCF, increasing the risk of severe complications such as pneumocephalus, CSF leakage, meningitis, and secondary infections. 4 5 6 For this reason, surgical intervention becomes critical in cases where these risks are apparent or where patients experience significant symptoms.

Two of the three patients in this study (Cases 1 and 2) underwent craniotomies for cyst removal and defect repairs using fascia lata or bone cement. These materials provided a durable structural barrier, restoring the integrity of the skull base and reducing the risk of future complications. Overall, Cases 1 and 2 show that this surgery is a safe and effective treatment for TMJ cysts with intracranial extension.

However, surgical intervention may not be the best course of action for every patient. For a patient with poor functional status, significant comorbidities, severe neurological deficits, or advanced age with low-performance scores, TMJ aspiration can be considered for management. As shown in Case 3, some patients may experience resolution of their symptoms with TMJ aspiration and tailored antibiotic therapy. Of note, this management approach requires close patient follow-up and monitoring to promptly identify and address any signs of progression or complications. Although there are no official guidelines available for postaspiration surveillance of this rare presentation, we would recommend serial imaging with CT of the temporal bone with and without contrast at a frequency dependent upon a given patient's symptoms.

### Presenting Symptoms and Outcomes

The presenting symptoms of TMJ cysts with intracranial extension are often nonspecific, reflecting the rarity and variable nature of these lesions. 1 2 3 4 5 6 In this study, all three patients presented with TMJ-related pain, but their associated symptoms varied widely, ranging from localized otalgia and headache to cranial nerve deficits and syncope. On imaging, there was evidence of chronic degenerative TMJ erosion in all three patients. However, only one patient (Case 2) noted chronic TMJ pain prior to the onset of this acute presentation. These cases highlight the importance of individualized treatment planning based on the patient's clinical presentation, imaging findings, and overall health status.

Case 1 exemplifies the more systemic manifestations of TMJ cysts with intracranial extension. This patient's dizziness, syncope, and facial weakness were unusual presenting symptoms that initially raised concerns for a more widespread or systemic neurological process. Imaging and surgical pathology ultimately confirmed the diagnosis of a benign synovial cyst, emphasizing the importance of considering TMJ cysts in the differential diagnosis of patients with atypical cranial symptoms. Most of this patient's presenting symptoms had resolved within 1 month after her surgery.

In contrast, Case 2 presented with more localized symptoms of TMJ pain and headache, which are more typical of these lesions. Imaging findings of cystic communication with the MCF and chronic bony erosion reinforced the need for surgical intervention, leading to a resolution of the patient's symptoms.

Case 3 presented a unique scenario in which conservative management was chosen. Despite the presence of purulence and bony erosion, this patient experienced resolution of symptoms with antibiotic therapy alone. However, this approach carries inherent risks, particularly in cases with significant bony defects or intracranial extension. Regular follow-up is crucial to ensure that symptoms do not recur and that no further complications arise.

### Clinical Implications and Future Directions

The findings of this study have several important implications for the diagnosis and management of TMJ cysts with intracranial extension. First, advanced imaging techniques, particularly MRI with DWI, are indispensable for accurately diagnosing these lesions and differentiating them from abscesses. These imaging findings should be interpreted in the context of clinical and laboratory data to guide management decisions.

Second, the role of surgical intervention in cases with bony erosion or symptomatic cysts is well supported by the outcomes of this study. Surgical repair using materials such as fascia lata and bone cement provides a durable structural barrier, preventing future complications and improving patient outcomes. However, further research is needed to evaluate the long-term outcomes of different repair techniques and materials.

Third, the potential for conservative management in selected cases warrants further exploration. As seen in Case 3, patients with significant comorbidities or advanced age with low-performance scores may choose antibiotic therapy and TMJ aspiration for symptom resolution as an alternative to surgical intervention. The risks of this approach must be carefully weighed against the potential benefits. Factors such as the cyst's size, the extent of intracranial extension, symptom severity, and the risk of complications like infection or neurological deterioration must all be carefully considered.

Finally, the complexity and rarity of TMJ cysts with intracranial extension demand a multidisciplinary approach that leverages the unique strengths of Otolaryngology, Neurosurgery, and OMFS. Each specialty provides critical expertise that ensures accurate diagnosis, effective surgical management, and comprehensive postoperative care. The seamless integration of these disciplines not only optimizes clinical outcomes but also enhances the patient experience by addressing the full spectrum of symptoms and concerns associated with these challenging lesions. Future studies may further refine this collaborative model, promoting even better outcomes for patients with this rare pathology.

## Conclusion

This study examines clinical presentations, diagnostic challenges, and possible treatments of TMJ cysts with MCF extension. Given the ability of these cysts to mimic intracranial abscesses, accurate diagnosis is required to guide appropriate clinical management. Key findings that point toward a cystic lesion, rather than an abscess, include continuity with the TMJ and an absence of water restriction on imaging. Without treatment, TMJ cysts with intracranial extension may predispose patients to dangerous complications such as pneumocephalus or secondary infections. Surgical intervention and defect repair, as performed in this study using fascia lata and bone cement, provided effective symptom relief and reduced the likelihood of future complications.
